# Application of Core–Shell Bimetallic Nanoparticles with Polydopamine-Assisted Nanogap in SERS-Based Lateral Flow Immunoassay of Prolactin

**DOI:** 10.3390/s26103064

**Published:** 2026-05-12

**Authors:** Kseniya V. Serebrennikova, Nadezhda S. Komova, Anatoly V. Zherdev, Boris B. Dzantiev

**Affiliations:** A.N. Bach Institute of Biochemistry, Research Center of Biotechnology of the Russian Academy of Sciences, Leninsky Prospect 33, Moscow 119071, Russia; ksenijasereb@mail.ru (K.V.S.); nad4883@yandex.ru (N.S.K.); zherdev@inbi.ras.ru (A.V.Z.)

**Keywords:** surface-enhanced Raman scattering (SERS), SERS nanotag, polydopamine-mediated internal nanogap, bimetallic Au^DTNB^@PDA^DTNB^@Ag nanoparticles, prolactin

## Abstract

The fabrication of SERS nanotags with efficient antibody loading and high signal enhancement remains a challenging task for combining surface-enhanced Raman spectroscopy (SERS) and lateral flow immunoassay (LFIA). In this study, bimetallic Au^DTNB^@PDA^DTNB^@Ag nanoparticles with a polydopamine (PDA)-based internal nanogap were synthesized and functionalized with anti-prolactin monoclonal antibodies to produce SERS nanotags. Here, polydopamine serves both as a spacer providing a nanogap between the core and the shell, and as a reaction layer to capture Raman reporter 5,5′-dithiobis(2-nitrobenzoic acid) (DTNB) within the nanogap. Regimes (conditions, protocols) for conjugating antibodies to Au^DTNB^@PDA^DTNB^@Ag were selected to preserve both the binding affinity for the target analyte and the Raman activity of the SERS nanotag. The SERS nanotag provides plasmonic absorption for visible colorimetric readout, as well as strong SERS signals for highly sensitive quantitative immunoassay. Measuring the Raman intensities of DTNB in the test zone after performing LFIA made it possible to determine prolactin with a detection limit of 0.2 ng/mL in the working range from 1 to 10 ng/mL. The achieved limit of detection was 10-fold lower than the LFIA coupled with colorimetric readout (4.7 ng/mL). The recoveries of prolactin from spiked serum samples were in the range of 70.2–82.6% with relative standard deviations of 2.3–6.8%. Overall, the Au^DTNB^@PDA^DTNB^@Ag nanotag demonstrated high stability, Raman activity, and specificity, indicating that the SERS nanotag with PDA-assisted internal nanogap is promising for use in SERS immunoassay of other target analytes.

## 1. Introduction

The integration of surface-enhanced Raman spectroscopy (SERS) with immunoassays has emerged as a promising approach for achieving highly sensitive quantitative detection of biomolecules [1,2,3,4]. In particular, the combination of SERS with lateral flow immunoassay (LFIA) offers an attractive platform combining rapid and simple point-of-care visual screening of LFIA with robust quantitative SERS readout [5,6,7]. This hybrid approach has gained widespread acceptance in clinical diagnostics, environmental monitoring, and bioanalytical applications, where specific detection and reliable quantification of trace analytes in complex biological environments are essential [8,9,10,11]. However, the practical implementation of LFIA combined with SERS readout relies heavily on the fabrication of robust SERS nanotags that can simultaneously provide strong Raman signals, stable optical properties, and efficient biorecognition [12,13,14].

One of the current challenges is the development of SERS nanotags that provide high Raman activity and are suitable for immunoassay in complex environmental conditions [15]. For example, the study [16] found that the sensitivity of SERS detection decreases with increasing molecular size of the antigen–antibody complex, which should be considered when developing SERS immunoassays. Traditional gold/silver nanospheres used in SERS, although effective as plasmonic enhancers, often suffer from signal instability, limited ability to load Raman reporters, and reduced efficiency upon surface modification [15]. Conjugation of plasmonic substrates with antibodies, which provide specific interaction with the target analyte, can further weaken the SERS signal due to surface blocking, increased interparticle distance, or disruption of electromagnetic “hot spots” [17,18]. Furthermore, ensuring the colloidal stability and reproducibility of SERS nanotags during LFIA in complex biological environments remains an ongoing challenge [19].

To overcome these limitations, various nanostructures have been proposed, including anisotropic gold/silver nanoparticles and bimetallic core–shell nanoparticles [20,21,22]. Despite the unique optical and electrical properties of sharp-edged nanoparticles, these nanostructures tend to lose their original complex structure at high temperatures [23,24]. In contrast, bimetallic core–shell nanoparticles are considered more stable and promising substrates for SERS spectroscopy [25,26,27]. A particular advantage of introducing nanogaps between the core and shell is the enhancement of electromagnetic fields in these confined areas and the formation of protected sites for Raman reporter molecules [28,29,30]. Thus, the stability of the SERS nanotag in the environment and its spectral homogeneity lead to the widespread use of core–shell substrates in SERS immunoassays [31,32,33,34]. Strategies for forming gaps with controlled size and optical properties include the use of silica, polymers, aptamers, and other small molecules as core–shell spacers [29,35,36]. Polydopamine (PDA) has proven to be a versatile material due to its strong adhesive properties, biocompatibility, and ability to act as both a spacer layer and a functional template for chemical modification [37,38,39,40]. Nanogaps created by PDA not only facilitate the formation of reproducible “hot spots” but also enable the efficient insertion of Raman reporters, ensuring their stability and protection from desorption and environmental influences [38].

Despite a number of advances in the development of SERS nanotags, the establishment of a universal structure that meets all the requirements of immunoassay and ensures high sensitivity for the determination of biomarkers in clinically important ranges remains an urgent task. Therefore, this study focuses on combining existing approaches and developing Au^DTNB^@PDA^DTNB^@Ag comprising a core–shell structure, an additional internal polydopamine-based nanogap, and a reporter molecule embedded within the gap for use as multifunctional SERS nanolabels in immunoassay. In the first step, spherical gold nanoparticles modified with the DTNB reporter molecule were prepared and then coated with a PDA layer via oxidative polymerization of dopamine. Since PDA not only acts as a spacer between the core and shell but also as a reactive layer with multiple catecholamine, amine, and imine functional groups, the latter property of polydopamine was used to increase the loading of the SERS substrate with a Raman-active label. In the final step, the nanoparticles were coated with a silver shell to obtain Au^DTNB^@PDA^DTNB^@Ag. Furthermore, several antibody conjugation methods were explored to preserve both antigen-binding activity and the integrity of the Raman signal of the SERS nanotag. The proposed SERS nanotag was tested in a quantitative SERS-LFIA assay, using prolactin as a model analyte. Prolactin is a clinically significant hormone, with abnormal levels associated with various endocrine disorders, requiring sensitive and accurate detection methods [41,42]. Integration of SERS nanotags into the conventional LFIA format improves analytical sensitivity compared to traditional colorimetric immunoassays while maintaining the ease of use necessary for practical application. Thus, this work aims to overcome key challenges in the development of SERS-LFIA and demonstrate a robust SERS platform for the sensitive detection of prolactin, which has broader implications for the analysis of other clinically relevant biomarkers.

## 2. Materials and Methods

### 2.1. Reagents and Materials

Gold (III) chloride hydrate (HAuCl_4_ × 6H_2_O), sodium citrate, L-ascorbic acid, sodium azide, bovine serum albumin (BSA), 5′-dithio-bis(2-nitrobenzoic acid) (DTNB), mercaptobenzoic acid, Tris, Triton X-100. Methoxy PEG Thiol (mPEG-SH, 5 kDa) was purchased from Abbexa (Cambridge, UK). Dopamine hydrochloride was from Shanghai Aladdin Biochemical Technology Co., Ltd. (Shanghai, China). OPSS-PEG-NHS ester was from Creative PEGWorks (Chapel Hill, NC, USA). The monoclonal antibodies (mAb) to prolactin (PRL), clones Pro-AE-001 (mAb1) and Pro-AE-002 (mAb2) were obtained from Fapon Biotech (Dongguan, China).

Recombinant human prolactin was from Fine Test (Wuhan, China). Luteinizing hormone (human) was purchased from MedChemExpess (Monmouth Junction, NJ, USA). Human alpha-fetoprotein (purified) was from Imtek (Moscow, Russia). Human chorionic gonadotropin (hCG) was acquired from Moscow Endocrine Plant (Moscow, Russia). This study used a lyophilized control serum “Clin Chem Control 1” prepared from human serum, which was acquired from Sentinel (Milan, Italy). All other chemicals were of analytical grade and provided by Khimmed (Moscow, Russia). Nitrocellulose membranes (CNPC type) for manufacturing lateral flow tests were from Advanced Microdevices (MDI, Ambala Cantt, India). Absorption pad CFSP223000 was from Millipore (Bedford, MA, USA). L-P25 plastic support was purchased from Advanced Microdevices (MDI, Ambala Cantt, India).

### 2.2. Synthesis of Core–Shell Nanoparticles

Core–shell Au^DTNB^@PDA^DTNB^@Ag nanoparticles were prepared stepwise using modified techniques [38,43,44,45]. At the first stage, Au nanoparticles (AuNPs) were obtained by reducing chloroauric acid with sodium citrate [43]. To do this, 1.3 mL of 1% (*w*/*v*) sodium citrate was added to 100 mL of a boiling 0.01% (*w*/*v*) HAuCl_4_ solution and kept for 15 min at 100 °C. After this, the AuNPs were cooled and stored at a temperature of 4–6 °C. The resulting AuNPs were washed by centrifugation at 7500× *g* for 15 min and re-dissolved in the same volume in deionized water.

In the next step of synthesis, 10 μL of a 1 mM ethanol solution of DTNB was added to 10 mL of AuNPs and incubated at RT for 1 h. The Au^DTNB^ nanoparticles was then centrifuged at 5500× *g* for 15 min and redissolved in water.

The formation of a polydopamine layer on the surface of gold nanoparticles was achieved by oxidative polymerization of dopamine for 1 h at RT [38,44]. In this study, two final concentrations of dopamine in solution were tested: 0.02 and 0.2 mg/mL (the stock dopamine solution was prepared in Tris buffer, pH 8.6). The resulting Au^DTNB^@PDA1 and Au^DTNB^@PDA2 nanoparticles, obtained by polymerization of 0.02 mg/mL and 0.2 mg/mL dopamine, respectively, were washed with deionized water by centrifugation at 5500× *g* for 15 min.

At the next stage, the Au^DTNB^@PDA nanoparticles were modified with a DTNB reporter molecule. For this, 10 μL of a 1 mM DTNB solution was added to 10 mL of Au^DTNB^@PDA nanoparticle dispersion and incubated for 30 min at room temperature with constant stirring.

To obtain Au^DTNB^@PDA^DTNB^@Ag core–shell nanoparticles, a modified method was used [45], in which 100 μL of 0.1 M ascorbic acid was added to 10 mL of Au^DTNB^@PDA^DTNB^. Then, 200 μL of 10 mM AgNO_3_ was added dropwise to the reaction mixture, and the mixture was stirred for 1 h. After this, the core–shell Au^DTNB^@PDA^DTNB^@Ag nanoparticles were centrifuged at 5500× *g* and re-dissolved in water.

Core–shell Au^DTNB^@Ag nanoparticles were prepared using a similar procedure, excluding the step of formation the polydopamine layer.

### 2.3. Characterization of Nanoparticles

The obtained nanoparticles were characterized using absorption spectroscopy, dynamic light scattering, and transmission electron microscopy (TEM). Absorption spectra were recorded using a UV-2450 spectrophotometer (Shimadzu, Kyoto, Japan). The hydrodynamic diameters of the nanoparticles were determined by dynamic light scattering on a Zetasizer Nano ZSP (Malvern, UK). To obtain micrographs using a JEM-100C electron microscope (Jeol, Tokyo, Japan), solutions of nanoparticles were applied to grids (300 mesh) coated with a polyvinyl formalin backing film dissolved in chloroform. The resulting images were analyzed using Image Tool software, version 3.0 (San Antonio, TX, USA).

### 2.4. Estimation of Enhancement Factor

The Raman enhancement properties of Au^DTNB^, Au^DTNB^@Ag, Au^DTNB^@PDA1^DTNB^@Ag, and Au^DTNB^@PDA2^DTNB^@Ag nanoparticles were evaluated using enhancement factor (EF). The EF was calculated according to

(1) AEF=ISERSCSERSIRamanCRaman

where $I_{SERS}$ and $I_{Raman}$ are the intensities of the characteristic DTNB Raman band at 1335 cm^−1^ obtained under SERS and normal Raman conditions, respectively, and $C_{SERS}$ and $C_{Raman}$ are the corresponding concentrations of DTNB. The Raman spectrum of DTNB was recorded for a 0.1 M solution.

### 2.5. Preparation of SERS Nanotags

For covalent immobilization, the antibodies were pre-activated by adding 20 μL of an aqueous solution of OPSS-PEG-NHS (2 mg/mL) to 1 mL of mAb1 (100 μg/mL in 0.1 M carbonate buffer, pH 9.6) and incubating for 45 min. The mixture was then centrifuged using Amicon 30 K microcentrifuge tubes at 10,000× *g* and 4 °C for 15 min in PBS. The centrifugation procedure was repeated three times. Next, the modified antibodies were added to 1 mL of Au^DTNB^, Au^DTNB^@Ag and Au^DTNB^@PDA^DTNB^@Ag nanoparticle solutions to a final concentration of 15 μg/mL and incubated for 1 h at RT. Finally, 20 μL of an aqueous solution of mPEG-SH (5 mg/mL) were added to the resulting mixture, the mixture was left for another 30 min, and unbound components were removed by centrifugation twice at 5000× *g* and 4 °C for 15 min. The pellet was resuspended in PBS and stored at 4 °C.

For physical adsorption of mAb1, 1 mL of Au^DTNB^@PDA^DTNB^@Ag nanoparticle solutions was adjusted to pH 8.5 with 0.2 M Na_2_CO_3_, after which mAb1 was added to a final concentration of 15 μg/mL. The mixture was incubated for 2 h with stirring at RT, then 20 μL of mPEG-SH (5 mg/mL) were added and incubated for another 30 min. The resulting conjugate was precipitated by centrifugation at 5000× *g* for 15 min at 4 °C, dissolved in 10 mM PBS, and stored at 4 °C.

### 2.6. Fabrication of LFIA Test Strips

LFIA test strips were prepared using a CNPC nitrocellulose membrane CNPC-SS12 with a pore size of 15 μm and an AP-045 absorbent pad, attaching them to an L-P25 plastic support. The test line was formed by applying the monoclonal antibody mAb2 at concentrations of 0.25, 0.5, and 1 mg/mL and in phosphate-buffered saline (PBS) using an IsoFlow dispenser (Imagene Technology, Lebanon, NH, USA) at a flow rate of 1 μL/cm onto the nitrocellulose membrane and drying for 2 h at 37 °C. Then, the prepared LFIA test strips were cut into 4-mm-wide test strips using an Index Cutter-1 guillotine cutter (A-Point Technologies, Gibbstown, NJ, USA) and stored in a plastic bag with a desiccant at room temperature.

### 2.7. Procedure of Conventional LFIA with Colorimetric Readout and SERS-LFIA of Prolactin

The LFIA for prolactin determination involved pre-adding 40 µL of standard solutions containing prolactin at concentrations of 0.15–1000 ng/mL to the wells of a microplate. Then, the Au@PDA1^DTNB^@Ag ^DTNB^ -mAb1 was added to the wells in a volume varying from 0.5 to 1.5 μL, after which the test strips were vertically immersed in the solution.

The results of the conventional colorimetric LFIA were recorded using a CanoScan LiDE 90 scanner (Canon, Tokyo, Japan). For SERS analysis, after the test strips had completely dried, SERS spectra were recorded at 10 points along the test line using a Raman spectrometer with an NS200 microscope (Nanoscope Systems, Daejeon, Republic of Korea). All SERS spectra were obtained under identical conditions using the following settings: excitation laser—785 nm, laser power—20 mW, exposure time—20 s, objective—20× (NA = 0.46).

The dependences of the staining intensity (colorimetric signal) or the intensity of the characteristic band of DTNB at 1335 cm^−1^ in the SERS spectrum (Raman signal) in the analytical zone on the antigen concentration in the sample were approximated using the Origin version 9.0 software (OriginLab, Northampton, MA, USA) using a 4-parameter sigmoid function. The limit of detection was calculated as the mean signal value for the prolactin-free sample plus three times its standard deviation.

## 3. Results and Discussion

### 3.1. Synthesis and Characterization of Bimetallic Core–Shell SERS Nanotags

Core–shell Au^DTNB^@PDA^DTNB^@Ag nanoparticles with an internal nanogap were synthesized in a stepwise manner, including the preparation of citrate-stabilized AuNPs, adsorption of the DTNB Raman reporter, deposition of a polydopamine (PDA) layer, and subsequent growth of an outer silver shell. For comparison, Au^DTNB^@Ag nanoparticles without an intermediate PDA layer were also prepared.

**Figure 1:**
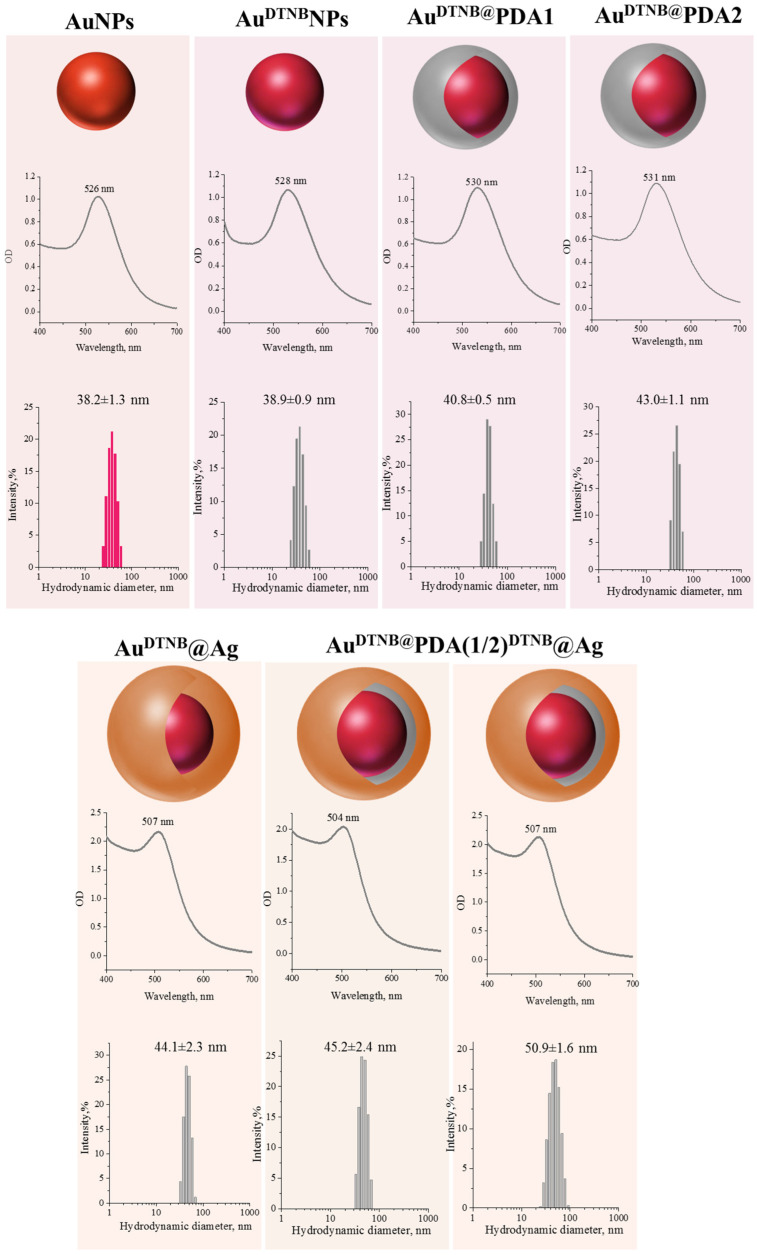
Absorption spectra and histograms of nanoparticle size distribution registered by DLS registered for AuNPs, Au^DTNB^ NPs, Au^DTNB^@PDA1 NPs, Au^DTNB^@PDA2 NPs, Au^DTNB^@Ag NPs, Au^DTNB^@PDA1^DTNB^@Ag and Au^DTNB^@PDA2^DTNB^@Ag.

The formation of nanostructures at each stage was monitored using absorption spectroscopy, the results of which are presented in Figure 1. The initial AuNPs exhibited a characteristic localized surface plasmon resonance (LSPR) band at approximately 526 nm, indicating the formation of well-dispersed spherical nanoparticles. After modification with DTNB, only a slight red shift and minor broadening of the absorption band were observed, confirming successful functionalization without aggregation. Coating with polydopamine using two different concentrations of dopamine stock solution resulted in a slight red shift of the localized surface plasmon resonance maximum to 530 nm without additional band broadening, confirming the formation of a thin polymer shell and maintaining the stability of the nanoparticles. After deposition of the silver shell, the absorption spectra of Au^DTNB^@Ag and Au^DTNB^@PDA^DTNB^@Ag nanoparticles shifted to shorter wavelengths and broadened slightly, confirming the formation of a silver shell on the nanoparticle surface.

Dynamic light scattering (DLS) measurements demonstrated a gradual increase in the hydrodynamic diameter after each modification step, as shown in Figure 1. AuNPs displayed the smallest size, followed by a slight increase after DTNB adsorption, a more pronounced increase after PDA coating, and the largest size after Ag shell formation. Notably, Au^DTNB^@PDA^DTNB^@Ag nanoparticles exhibited a larger hydrodynamic diameter than AuDTNB@Ag, consistent with the presence of the PDA interlayer. All nanoparticle systems demonstrated good colloidal stability, as indicated by relatively narrow size distributions and the absence of significant aggregation.

**Figure 2:**
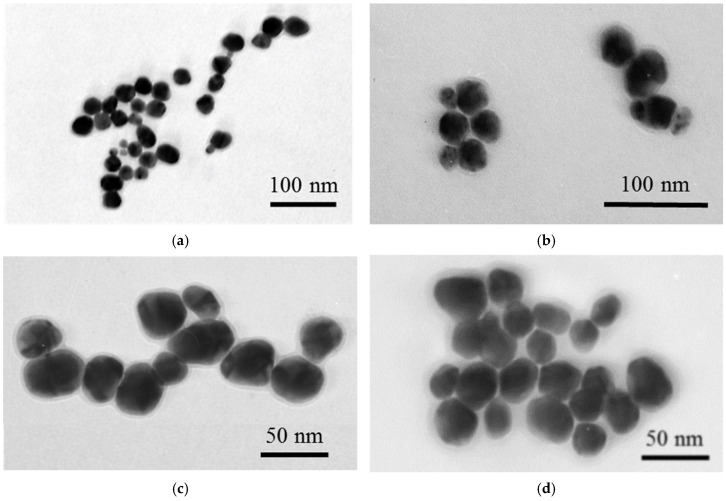
TEM images of (**a**) AuNPs, (**b**) Au^DTNB^@Ag NPs, (**c**) Au^DTNB^@PDA1^DTNB^@Ag and (**d**) Au^DTNB^@PDA2^DTNB^@Ag.

Transmission electron microscopy (TEM) analysis confirmed the morphology and structure of the synthesized nanoparticles (Figure 2). The AuNPs were predominantly spherical with a narrow size distribution and an average diameter of 30.3 ± 2.6 nm. After Ag deposition, Au^DTNB^@Ag nanoparticles exhibited a quasi-spherical shape with an Ag layer forming surrounding the Au core. The average diameter of Au^DTNB^@Ag was estimated to be 41.5 ± 5.1 nm. In the case of Au^DTNB^@PDA1^DTNB^@Ag, the average nanoparticle diameter increased slightly to 42.6 ± 5.3 nm, indicating the formation of an ultrathin intermediate PDA layer. Increasing the initial dopamine concentration during the synthesis of Au^DTNB^@PDA^DTNB^@Ag resulted in a slight increase in the intermediate PDA layer between the core and shell. The average diameter of Au^DTNB^@PDA2^DTNB^@Ag was 43.1 ± 4.9 nm. Silver deposition on Au^DTNB^@PDA^DTNB^ likely initially occurs through island nucleation on the PDA surface, followed by coalescence into a quasi-continuous layer, as indicated by TEM data. Such controlled growth is important for formation of uniformly distributed plasmonic hotspots, which directly impacts the reproducibility of the SERS signal.

Although the spatial resolution of conventional TEM used in this study was insufficient to directly visualize the ultrathin PDA interlayer, the systematic increase in nanoparticle diameter after PDA deposition confirms the formation of a PDA-assisted internal nanogap between Au core and Ag shell. Based on the difference in average particle diameters between Au^DTNB^@Ag and Au^DTNB^@PDA^DTNB^@Ag nanoparticles, the effective thickness of the PDA layer was estimated to be approximately 0.5–0.8 nm. This indirect estimate is further supported by the pronounced increase in SERS intensity observed for Au^DTNB^@PDA1^DTNB^@Ag (see Section 3.2), which is consistent with the nanogap-mediated electromagnetic field enhancement previously reported for PDA-containing core–shell systems [38,44].

### 3.2. Comparison of Raman Activity of SERS Nanotags

The SERS performance of the nanostructures was evaluated using DTNB as a Raman reporter. DTNB is a common reporter molecule due to its large Raman scattering cross section without fluorescence interference and the formation of metal-S covalent bonds with the surface of plasmonic nanoparticles [46,47].

SERS measurements were performed using a 785 nm excitation laser despite the localized surface plasmon resonance (LSPR) maximum of the synthesized Au^DTNB^@PDA^DTNB^@Ag nanoparticles being near 504–507 nm. The wavelength corresponding to the LSPR maximum of the nanostructures is not necessarily the optimal excitation wavelength for SERS, especially in nanogap-enhanced core–shell nanoparticles. In such nanostructures, plasmonic coupling between Au and Ag shell generates localized electromagnetic hotspots within the nanogap, and the associated plasmonic modes can extend into the near-infrared range [48,49]. Furthermore, using excitation at 785 nm minimizes fluorescence background from biological matrices and nitrocellulose membranes, reduces photothermal effects, and improves signal stability [19,50,51], which is particularly important for LFIA coupled with SERS detection.

A commonly used measure of Raman enhancement properties is the enhancement factor, which is calculated as the ratio of the SERS intensity to the number of excited molecules [52,53,54]. In this study, the Raman enhancement properties of Au^DTNB^, Au^DTNB^@Ag, Au^DTNB^@PDA1^DTNB^@Ag, and Au^DTNB^@PDA2^DTNB^@Ag were evaluated using AEF (see Equation (1) in Section 2.4). For the AEF calculations (see Table 1), we used the most intense SERS signal (at 1335 cm^−1^) and the normal Raman peak (at 1340 cm^−1^) for 100 mM DTNB, assuming that the DTNB concentration (according to the synthesis protocol) was 10^−6^ M for Au^DTNB^, Au^DTNB^@Ag nanoparticles and 2 × 10^−6^ M for Au^DTNB^@PDA1^DTNB^@Ag, and Au^DTNB^@PDA2^DTNB^@Ag. The achieved AEF value for bimetallic Au^DTNB^@PDA2^DTNB^@Ag nanoparticles is comparable to the AEF values reported for nanogap-enhanced Raman nanotags based on Au@Au core/shell nanoparticles [52,55], DTNB-encoded satellite Fe_3_O_4_@Au [33] and Au@Ag@Ag nanorods [56].

Figure 2 shows the Raman spectra of DTNB obtained for SERS nanotags after application of the sample, containing prolactin, and standard LFIA procedure. SERS nanotags demonstrated characteristic peaks of DTNB at 1061 cm^−1^, 1335 cm^−1^, and 1557 cm^−1^, corresponding to C–N bending, C–N stretching vibration, and the symmetric stretching vibration of the nitro-group (NO_2_), respectively. The dominant peak at 1335 cm^−1^ was selected to evaluate the SERS performance of the nanotags and for quantitative SERS analysis.

**Figure 3:**
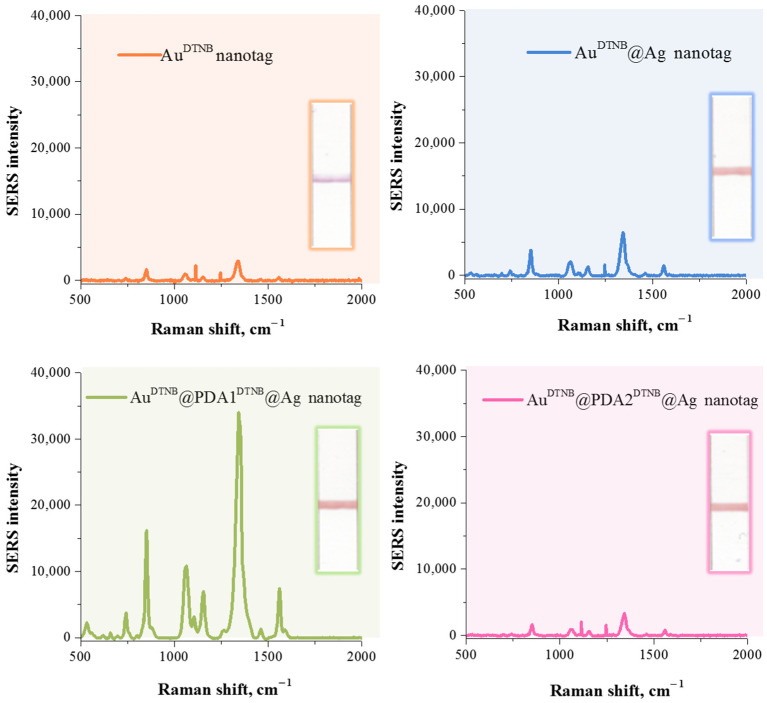
SERS spectra of DTNB and digital images of test strips obtained during LFIA in the presence of 1000 ng/mL, using Au^DTNB^, Au^DTNB^@Ag, Au^DTNB^@PDA1^DTNB^@Ag and Au^DTNB^@PDA2^DTNB^@Ag nanotags.

As can be seen from Figure 3, DTNB-modified gold nanoparticles exhibited relatively weak Raman signals due to limited electromagnetic field enhancement. Encapsulation of the Raman reporter with a silver shell resulted in a two-fold increase in the Raman signal due to the generation of a strong electric field in the gap between the adjacent gold surface and the silver layer. Synthesis of SERS nanotags with the Au^DTNB^@PDA^DTNB^@Ag structure resulted in the formation of both a nanogap between the gold core and the silver shell, and a reactive layer that allows the immobilization of reporter molecules within the nanogap. However, the thickness of the polydopamine layer significantly affects the SERS signal (see Figure 3, Raman spectra of Au^DTNB^@PDA1^DTNB^@Ag and Au^DTNB^@PDA2^DTNB^@Ag). A comparison of the SERS spectra of nanotags obtained by polymerizing dopamine with final concentrations of 0.02 mg/mL and 0.2 mg/mL in solution revealed that a significant increase in SERS intensity was observed at the lower monomer concentrations. The enhanced SERS signal in this case is explained by improved retention of DTNB molecules near the metal surface and changes in the local dielectric environment.

However, the SERS signal of Au^DTNB^@PDA2^DTNB^@Ag nanotag decreased significantly and became lower than that of Au^DTNB^@Ag. This observed effect may be due to the fact that the polymer layer thickness exceeded a critical threshold, preventing the 785 nm excitation laser from penetrating the polymer layer to detect the Raman signal of DTNB. These data are consistent with a study that found that the thickness of the polydopamine shell plays a significant role in the SERS performance of core–shell systems [44].

Thus, the comparison of SERS nanotags showed that AuDTNB@Ag nanoparticles without a PDA interlayer have a lower SERS intensity, which emphasizes the important role of polydopamine in the formation of nanogaps. Furthermore, the Michael addition reactions of quinone groups in PDA with nucleophilic thiols [57] allow saturation of the nanogaps with reporter molecules. Therefore, the Au^DTNB^@PDA1^DTNB^@Ag nanotag providing the highest SERS activity was applied for further research.

**Figure 4:**
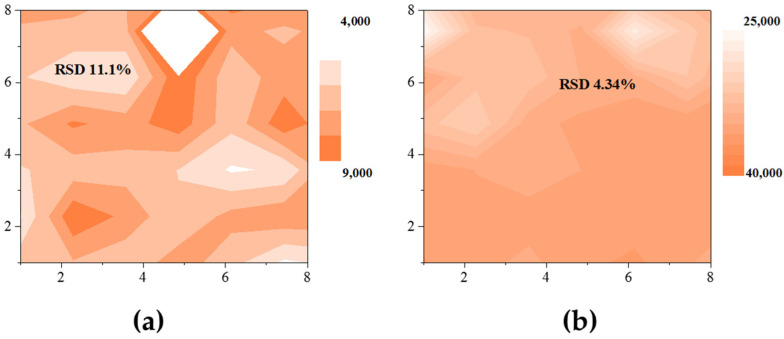
Raman mapping images (8 × 8 counts) of test strips obtained after SERS-LFIA using (**a**) Au^DTNB^@Ag-mAb and (**b**) Au^DTNB^@PDA^DTNB^@Ag-mAb conjugates for concentration of prolactin of 1000 ng/mL.

An assessment of the spatial distribution and comparison of the SERS signal reproducibility in the LFIA strip test zone obtained for Au^DTNB^@Ag and Au^DTNB^@PDA1^DTNB^@Ag nanotags revealed that Au^DTNB^@PDA1^DTNB^@Ag nanoparticles provide the most uniform SERS signal distribution (Figure 4). Statistical analysis of the mapping data revealed a relative standard deviation (RSD) of 4.34% for Au^DTNB^@PDA1^DTNB^@Ag versus 11.1% for Au^DTNB^@Ag nanotag. Therefore, the Au^DTNB^@PDA1^DTNB^@Ag nanotag, providing the highest SERS activity and high SERS signal reproducibility, was applied for further research.

**Figure 5:**
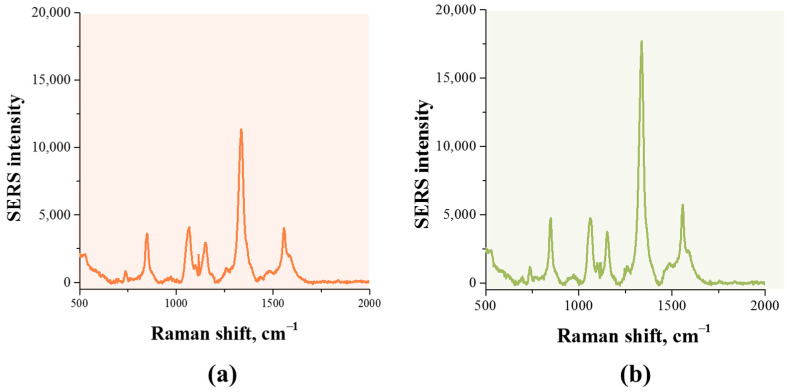
SERS spectra of DTNB obtained for SERS-LFIA of prolactin using Au^DTNB^@PDA^DTNB^@Ag conjugates with mAb1 immobilized by physical adsorption (**a**) and covalent binding (**b**).

Finally, two common antibody immobilization strategies were evaluated to ensure the preservation of SERS activity. As shown in Figure 5, high Raman signal intensity was observed for the Au^DTNB^@PDA1^DTNB^@Ag nanotag obtained by covalent immobilization of antibodies via cross-linking agent (OPSS-PEG-NHS). The presence of a PEG spacer between the surface of the nanoparticle and the antibody helps to increase the availability of antigen binding by the antibody and reduce steric hindrances. In contrast, physical adsorption of antibodies on the nanoparticle surface resulted in a significant decrease in SERS activity. The observed decrease in Raman signal is likely due to the random arrangement of antibodies on the nanoparticle surface and the formation of a thick protein shell, which partially blocked hotspots and reduced the accessibility of DTNB molecules to the laser excitation. These results indicate that covalent immobilization is a preferred approach for obtaining stable and highly active SERS nanotags suitable for immunoassays.

### 3.3. Optical and Raman Performance of SERS-LFIA

**Figure 6:**
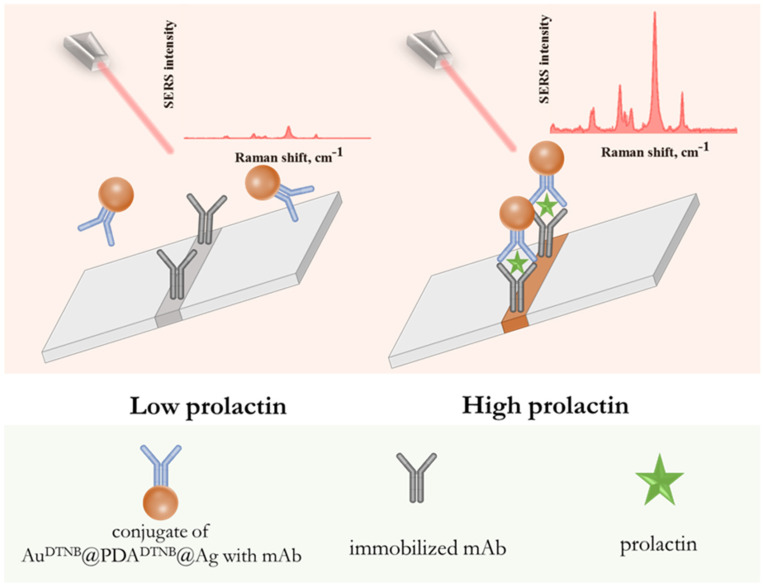
Principle of SERS-LFIA of prolactin using Au^DTNB^@PDA^DTNB^@Ag nanotag.

The analytical performance of the developed SERS-LFIA was evaluated using a sandwich assay format illustrated in Figure 6. In this configuration, prolactin molecules in the sample bind to anti-prolactin monoclonal antibodies immobilized on the surface of Au^DTNB^@PDA^DTNB^@Ag SERS nanotags, forming antigen–antibody complexes. These complexes migrate along the test strip via capillary forces and are captured in the test zone by a secondary immobilized antibody specific to prolactin, resulting in the formation of a sandwich structure. The accumulation of nanotags in the test zone generates both a visible colorimetric signal due to plasmonic absorption and a SERS signal originating from DTNB molecules localized within the nanogap. While the colorimetric signal can be assessed visually or by measuring the intensity of the colored test line, quantitative detection is achieved by recording the Raman signal intensity of DTNB.

**Figure 7:**
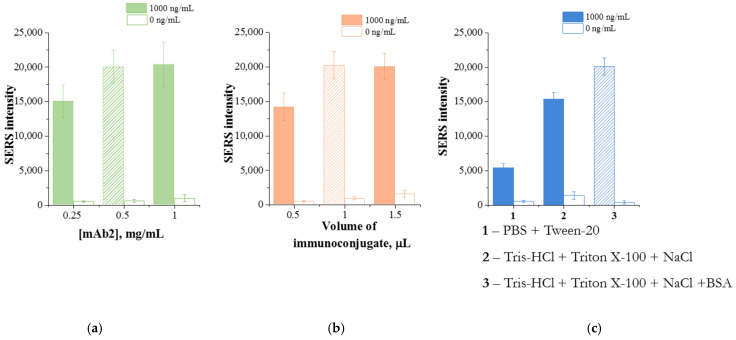
Raman intensities of DTNB obtained after performing LFIA in the presence of 100 ng/mL prolactin and in the absence of the analyte under varying the following conditions: the concentration of immobilized mAb2 (**a**), volume of immunoconjugate (**b**) and running buffer (**c**). The selected conditions are marked in the figures by a shaded area.

To reduce non-specific adsorption and increase the detection sensitivity of SERS-LFIA, the concentration of immobilized antibodies on the nitrocellulose membrane, the volume of the SERS nanotag, and the composition of the running buffer were optimized (Figure 7). The following optimal conditions were selected for SERS-LFIA: 0.5 mg/mL of immobilized mAb2 in the test zone, 1 μL of the SERS nanotag, and a running buffer consisting of Tris-HCl, 1% Triton X-100, 1% NaCl, and 1% BSA, pH 8.5.

**Figure 8:**
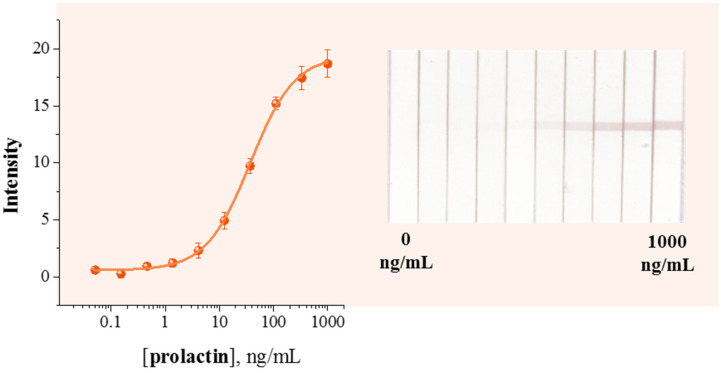
Calibration curves for prolactin detection obtained by colorimetric LFIA using Au^DTNB^@PDA^DTNB^@Ag-mAb conjugate. Inserts: digital images of LFIA test strips obtained for 0, 0.15, 0.46, 1.37, 4.11, 12.3, 37.3, 111, 333 and 1000 ng/mL of prolactin. The error bars shown in the graph were estimated based on three repeats of analysis.

The performance of the SERS-LFIA was compared with that of a conventional colorimetric LFIA using the same SERS nanotag but relying solely on optical signal readout. Figure 8 shows the calibration dependence of the test zone staining intensity on the prolactin concentration and the corresponding digital images of the test strips after passing prolactin in the concentration range from 0 to 1000 ng/mL. Colorimetric readout of the LFIA results allows the determination of prolactin in a working concentration range from 10.1 to 136.7 ng/mL with a calculated detection limit of 4.7 ng/mL. The colorimetric assay enabled detection of prolactin based on the intensity of the test line; however, its sensitivity was limited by the intrinsic optical contrast.

**Figure 9:**
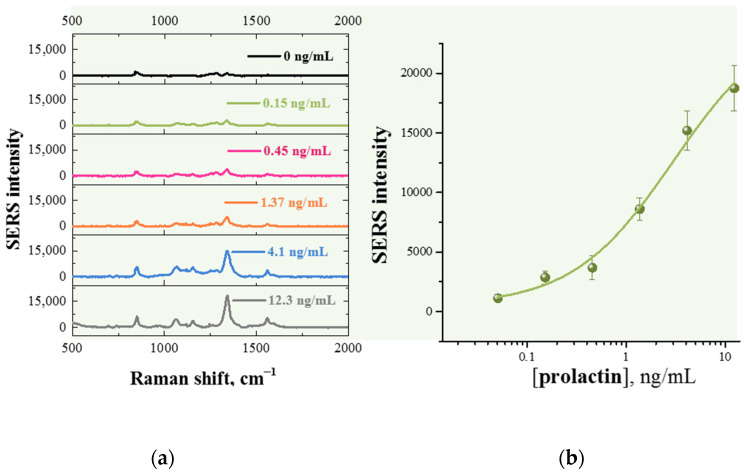
(**a**) SERS spectra of DTNB measured in the test zone of LFIA after application of different concentrations of prolactin and (**b**) calibration curve of SERS intensity at 1335 cm^−1^ as a function of the logarithm of concentration of prolactin obtained using Au^DTNB^@PDA^DTNB^@Ag nanotag. The error bars shown in the graph were estimated based on three repeats of analysis.

Figure 9 shows the SERS spectra obtained in the test zone of the strips after passing prolactin solutions in the concentration range of 0–12.3 ng/mL. The calibration dependence of the SERS signal intensity at 1335 cm^−1^, measured in the test zone, on the target analyte concentration showed a linear relationship in the range of 1–10 ng/mL, with a limit of detection (LOD) of 0.23 ng/mL for prolactin (Figure 9). As follows from Table 2, the detection limit obtained after SERS-LFIA is approximately an order of magnitude lower than the detection limit calculated after processing the colorimetric LFIA data.

**Figure 10:**
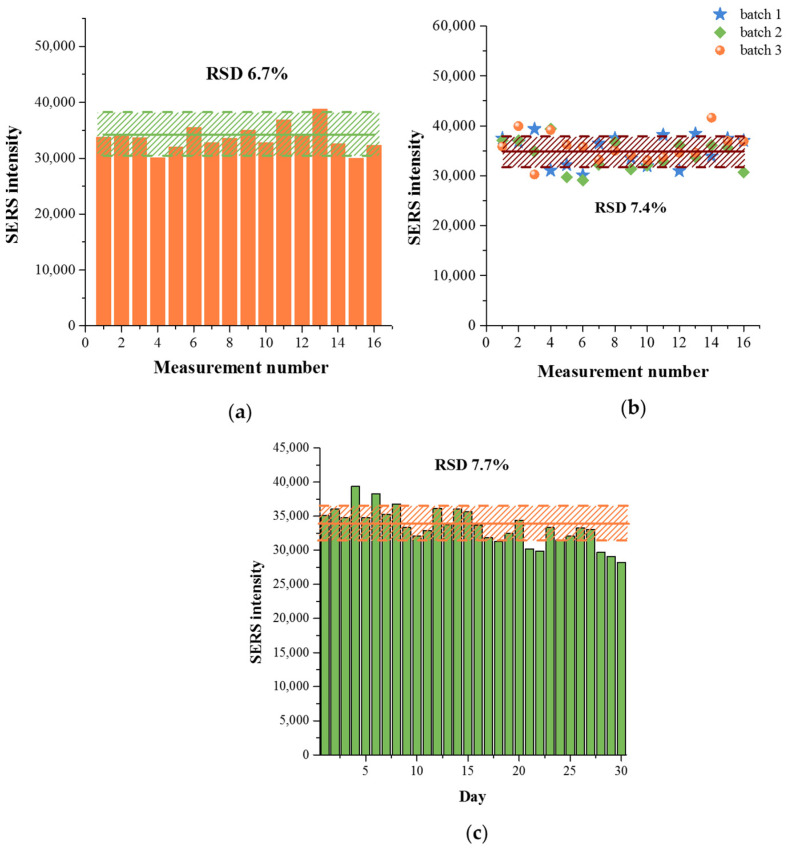
(**a**) SERS signal intensity at 1335 cm^−1^ measured at 16 different points on the test strip. (**b**) Inter-batch reproducibility of the SERS signal obtained for 1000 ng/mL prolactin. SERS spectra were measured at 16 different spots on the test strips using Au^DTNB^@PDA^DTNB^@Ag synthesized in three batches. (**c**) Stability of SERS nanotag when stored at 4 °C for 30 days. The shaded areas in all figures indicate RSDs.

The SERS signal homogeneity was investigated using Raman mapping of the test zone of the strips. Figure 10a shows the SERS intensities at 1335 cm^−1^ obtained for 1000 ng/mL prolactin at 16 different points. The SERS signal variability demonstrated good inter-strip reproducibility with a relative standard deviation (RSD) of 6.7%. To assess the batch-to-batch reproducibility of the nanoparticle synthesis, a solution containing 1000 ng/mL prolactin was tested using Au^DTNB^@PDA^DTNB^@Ag synthesized in three batches (Figure 10b). The RSD for these tests was 7.4%, confirming the good reproducibility of the Au^DTNB^@PDA^DTNB^@Ag synthesis and the developed SERS-LFIA method for prolactin. In addition, an assessment of the stability of the SERS signal during storage of test strips showed that the SERS nanotag retained its activity for 30 days of storage of test strips at 4 °C (Figure 10c).

The results clearly demonstrate that incorporation of SERS nanotags into the LFIA format significantly improves analytical sensitivity while maintaining the simplicity and rapidity of the lateral flow assay. The enhanced performance of the SERS nanotag is attributed to the presence of the PDA-assisted nanogap, which generates strong plasmonic “hot spots” and enables efficient amplification of the Raman signal. Consequently, the proposed SERS-LFIA platform provides a reliable and highly sensitive approach for quantitative detection of prolactin and holds promise for application to other clinically relevant biomarkers.

To evaluate the practical applicability of the SERS-LFIA based on Au^DTNB^@PDA^DTNB^@Ag nanotag, spiked serum samples were tested. As shown in Table 3, prolactin recovery rates ranged from 70.2% to 82.6%, with relative standard deviations (RSDs) ranging from 2.3% to 6.8% (*n* = 3). The slightly lower recovery observed in the prolactin-spiked serum samples (70.2–82.6%) compared to the standard range for clinical immunoassays (80–120%) may be due to the matrix effect of serum, which is a complex biological substance. Proteins, lipids, and other serum components can interfere with antigen–antibody interactions or partially block the surface of SERS nanotag. Furthermore, adsorption of serum proteins on the nanoparticle surface can partially shield plasmonic hot spots, resulting in a decrease in the intensity of the measured Raman signal. The recovery at the studied prolactin concentrations (3–5 ng/mL) is within the linear working range of the proposed SERS-LFIA (0.6–15.2 ng/mL), where excess antigen is not expected, indicating a low probability of signal suppression due to high analyte concentration and, consequently, the hook effect. However, the demonstrated ability of SERS-LFIA to determine prolactin in a clinically important range indicates the practical potential of the developed test system.

### 3.4. Specificity of SERS-LFIA

The specificity of the developed SERS-LFIA was evaluated by analyzing its response to potentially interfering biomolecules commonly present in clinical samples. A series of female hormones and protein biomarkers, including follicle-stimulating hormone (FSH), alpha-fetoprotein (AFP), luteinizing hormone (LH), human chorionic gonadotropin (hCG), and anti-Müllerian hormone (AMH), were tested under identical conditions at a concentration of 10 ng/mL. Prolactin at the same concentration was used as the target analyte for comparison. After completing the LFIA procedure, the Raman signal intensity of DTNB was measured in the test zone for each sample.

**Figure 11:**
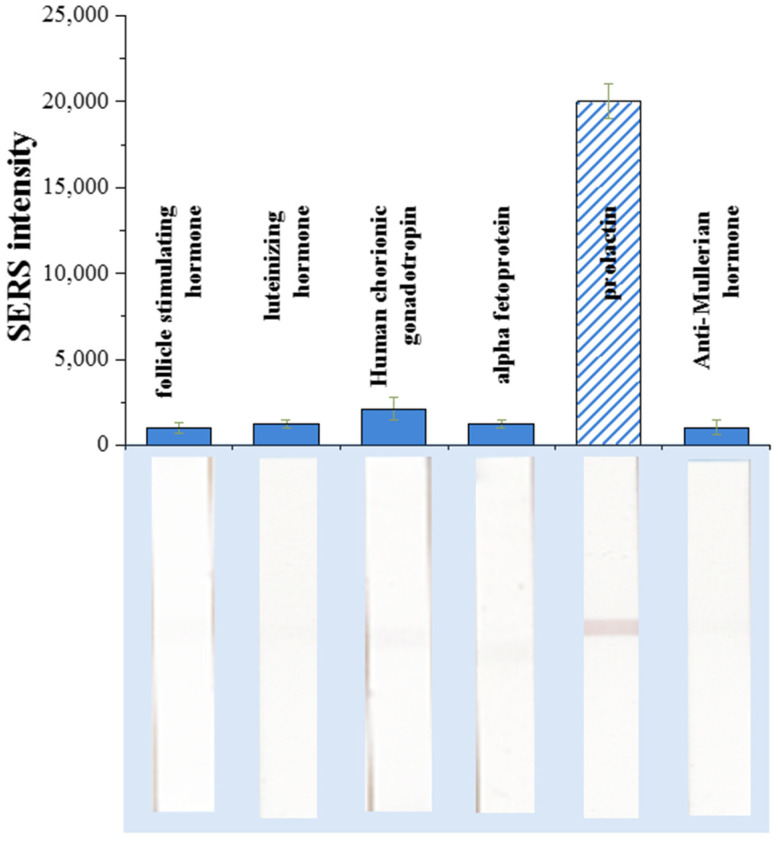
The specificity of the developed SERS-LFIA based on Au^DTNB^@PDA^DTNB^@Ag nanotag for prolactin detection in comparison with other hormones. The concentration of each hormone was 10 ng/mL.

As shown in Figure 11, a strong SERS signal was observed only in the presence of prolactin, whereas all other tested hormones produced signals at the background level. The negligible response obtained for FSH, AFP, LH, hCG, and AMH indicates minimal nonspecific binding of the SERS nanotags and confirms the high selectivity of the antibody–antigen interaction. This result demonstrates that the functionalization of Au^DTNB^@PDA^DTNB^@Ag nanoparticles with anti-prolactin monoclonal antibodies provides selective recognition of the target analyte even in the presence of structurally related or coexisting proteins.

Thus, the developed SERS-LFIA based on the Au^DTNB^@PDA^DTNB^@Ag nanotag enables the determination of prolactin in a clinically relevant range (normal prolactin levels in women range from 4.5 to 30 ng/mL) with limit of detection as low as 0.2 ng/mL [58]. Compared with existing prolactin determination methods listed in Table 4 [59,60,61,62], SERS-LFIA is a simple, quantitative, and reliable method with comparable analytical performance that also meets the requirements of clinical diagnostics.

## 4. Conclusions

This study combines recent advances in SERS nanotag design and demonstrates the successful development of bimetallic Au^DTNB^@PDA^DTNB^@Ag core–shell nanoparticles with a polydopamine-mediated internal nanogap. The activity of the SERS nanotag based on these nanogap-enhanced bimetallic nanoparticles and its effectiveness in immunoassays are confirmed by the high sensitivity of prolactin determination in the LFIA format coupled with quantitative SERS readout. The dual function of polydopamine as an internal nanogap between the gold core and silver shell and as a reaction layer for immobilizing the Raman reporter ensured both strong and reproducible SERS responses and nanotag stability. Furthermore, the chosen antibody conjugation strategy ensured the preservation of immunoreactivity without compromising signal intensity.

The developed SERS-LFIA platform combines the advantages of traditional LFIA, such as speed and ease of use, with the ability to quantitatively detect prolactin with a detection limit of 0.2 ng/mL. The achieved improvement in sensitivity of approximately one order of magnitude compared to conventional colorimetric LFIA demonstrates the effectiveness of the proposed SERS nanotag design for detecting low concentrations of biomarkers. The practical applicability of the SERS nanotag-based LFIA for biomarker analysis in complex biological samples was confirmed by testing spiked serum samples, yielding satisfactory recovery values. In summary, the proposed Au^DTNB^@PDA^DTNB^@Ag core–shell design offers a versatile solution for creating stable and highly active SERS nanotags, which can be easily adapted for the detection of a wide range of clinically relevant analytes.

## Figures and Tables

**Table 1:** The values of AEFs calculated for Au^DTNB^, Au^DTNB^@Ag, Au^DTNB^@PDA1^DTNB^@Ag, and Au^DTNB^@PDA2^DTNB^@Ag nanoparticles.

Sample	I_Raman, SERS_, a.u.	c_Raman, SERS_, M	AEF
Normal Raman	4175	10^−1^	n/a
Au^DTNB^	2938	10^−6^	1.8 × 10^5^
Au^DTNB^@Ag	6216	10^−6^	3.7 × 10^5^
Au^DTNB^@PDA1^DTNB^@Ag	33,883	2 × 10^−6^	2 × 10^6^
Au^DTNB^@PDA2^DTNB^@Ag	3378	2 × 10^−6^	2 × 10^5^

**Table 2:** Analytical performance of conventional LFIA and SERS-LFIA of prolactin using Au^DTNB^@PDA^DTNB^@Ag -mAb conjugate.

LFIA	Detection limit, ng/mL	4.7
	Linear range, ng/mL	10.1–136.7
SERS-LFIA	Detection limit, ng/mL	0.2
	Linear range, ng/mL	0.6–15.2

**Table 3:** Recovery rates for detection of prolactin in spiked serum samples using developed SERS-LFIA. RSD values were calculated based on three separate tests.

Added, ng/mL	Found, ng/mL	Recovery, %	RSD, %
3	2.1	70.2	2.3
5	4.13	82.6	6.8

**Table 4:** Comparison of different detection methods of prolactin.

Method	Label	Limit of Detection, ng/mL	Working Range, ng/mL	Ref.
Time-resolved fluorescence immunoassay	Eu^3+^ chelate	0.35	0.1–1000	[59]
Surface-enhanced Raman spectroscopy	Quasi-spherical AgNPs	0.1	n/a	[60]
Electrochemical immunoassay	n/a	0.13	1–250	[61]
Fluorescence immunoassay	Fluorescently labeled magnetic beads	0.00014	0.0018–1.3	[62]
SERS-LFIA	Au^DTNB^@PDA^DTNB^@Ag	0.2	0.6–15.2	This study

## Data Availability

The original contributions presented in this study are included in the article. Further inquiries can be directed to the corresponding author.

## References

[1] Smolsky J., Kaur S., Hayashi C., Batra S.K., Krasnoslobodtsev A.V. (2017). Surface-Enhanced Raman Scattering-Based Immunoassay Technologies for Detection of Disease Biomarkers. Biosensors.

[2] Lin C., Li Y., Peng Y., Zhao S., Xu M., Zhang L., Huang Z., Shi J., Yang Y. (2023). Recent development of surface-enhanced Raman scattering for biosensing. J. Nanobiotechnol..

[3] Lin L.L., Alvarez-Puebla R., Liz-Marzan L.M., Trau M., Wang J., Fabris L., Wang X., Liu G., Xu S., Han X.X. (2025). Surface-enhanced Raman spectroscopy for biomedical applications: Recent advances and future challenges. ACS Appl. Mater. Interfaces.

[4] Ranasinghe J.C., Sanders S.K., Wang Z., Mehrani J., Wu W., Dimitrov E., Wang X., Minns A.M., Rossi R.M., Lindner S.E. (2026). Noise Management of Surface-Enhanced Raman Spectroscopy Using Two-Dimensional Materials. ACS Sens..

[5] Liu Z., Jiang H., Lv X., Lian S., Li X. (2025). Performance Enhancement of SERS-Based Lateral Flow Assays: Progress and Prospective. Anal. Chem..

[6] Sloan-Dennison S., O’Connor E., Dear J.W., Graham D., Faulds K. (2022). Towards quantitative point of care detection using SERS lateral flow immunoassays. Anal. Bioanal. Chem..

[7] Rey Gomez L.M., Alom K.M., Nadalini A., Hnit S.S.T., Clark B., Lyu N., Hirani R., Sloan-Dennison S., Laing S., Rathmell C. (2026). A SERRS-Based Lateral Flow Assay for Sensitive Detection of Mitochondrial DNA in Endometriosis Screening. ACS Sens..

[8] Liu W., Wang H., Zhong W., Zhang Y., Liu Y., Gao X., Yan M., Zhu C. (2025). The development and application of SERS-based lateral flow immunochromatography in the field of food safety. Microchim. Acta.

[9] Tran V., Walkenfort B., König M., Salehi M., Schlücker S. (2019). Rapid, Quantitative, and Ultrasensitive Point-of-Care Testing: A Portable SERS Reader for Lateral Flow Assays in Clinical Chemistry. Angew. Chem. Int. Ed..

[10] Heo B., Jung H.S. (2025). SERS-Driven Evolution of Lateral and Vertical Flow Assays in Medical Diagnostics. Biosensors.

[11] Xue R.-S., Dai J.-Y., Wang X.-J., Chen M.-Y. (2025). Research Progress of Surface-Enhanced Raman Scattering (SERS) Technology in Food, Biomedical, and Environmental Monitoring. Photonics.

[12] Zhang W., Jiang L., Piper J.A., Wang Y. (2018). SERS Nanotags and Their Applications in Biosensing and Bioimaging. J. Anal. Test..

[13] Liu H., Gao X., Xu C., Liu D. (2022). SERS Tags for Biomedical Detection and Bioimaging. Theranostics.

[14] Lenzi E., Jimenez de Aberasturi D., Liz-Marzán L.M. (2019). Surface-Enhanced Raman Scattering Tags for Three-Dimensional Bioimaging and Biomarker Detection. ACS Sens..

[15] Vasquez I., Xue R., Srivastava I. (2025). Surface-Enhanced Raman Scattering Nanotags: Design Strategies, Biomedical Applications, and Integration of Machine Learning. WIREs Nanomed. Nanobiotechnol..

[16] Karn-Orachai K. (2021). Gap-Dependent Surface-Enhanced Raman Scattering (SERS) Enhancement Model of SERS Substrate–Probe Combination Using a Polyelectrolyte Nanodroplet as a Distance Controller. Langmuir.

[17] Chisanga M., Stuible M., Gervais C., L’Abbé D., Cass B., Bisson L., Pelletier A., Lord-Dufour S., Durocher Y., Boudreau D. (2022). SERS-based assay for multiplexed detection of cross-reactivity and persistence of antibodies against the spike of the native, P.1 and B.1.617.2 SARS-CoV-2 in non-hospitalised adults. Sens. Diagn..

[18] Hang Y., Wang A., Wu N. (2024). Plasmonic silver and gold nanoparticles: Shape- and structure-modulated plasmonic functionality for point-of-caring sensing, bio-imaging and medical therapy. Chem. Soc. Rev..

[19] Terzapulo X., Kassenova A., Bukasov R. (2024). Immunoassays: Analytical and Clinical Performance, Challenges, and Perspectives of SERS Detection in Comparison with Fluorescent Spectroscopic Detection. Int. J. Mol. Sci..

[20] Gao S., Guo Z., Liu Z. (2023). Recent Advances in Rational Design and Engineering of Signal-Amplifying Substrates for Surface-Enhanced Raman Scattering-Based Bioassays. Chemosensors.

[21] Chen X., Liu T., Chen W., Lin Z., Zhang Q., Zhou B., Zhou X. (2026). From nanotags to precision biomedicine: SERS-driven progress and innovation in tumor biomarker profiling, dynamic bioimaging, AI-enhanced diagnostics and therapy. Theranostics.

[22] Xie Y., Xu J., Shao D., Liu Y., Qu X., Hu S., Dong B. (2025). SERS-Based Local Field Enhancement in Biosensing Applications. Molecules.

[23] Attia Y.A., Altalhi T.A., Gobouri A.A. (2015). Thermal stability and hot carrier dynamics of gold nanoparticles of different shapes. Adv. Nanopart..

[24] Khanadeev V.A., Simonenko A.V., Grishin O.V., Khlebtsov N.G. (2023). One-Shot Laser-Pulse Modification of Bare and Silica-Coated Gold Nanoparticles of Various Morphologies. Nanomaterials.

[25] Awiaz G., Lin J., Wu A. (2023). Recent advances of Au@Ag core–shell SERS-based biosensors. Exploration.

[26] Wang C., Shi Y., Qin D., Xia Y. (2023). Bimetallic core–shell nanocrystals: Opportunities and challenges. Nanoscale Horiz..

[27] Cao E., Cao Y., Sun M. (2024). Surface Plasmonic Core–Shell Nanostructures in Surface Enhanced Raman Scattering and Photocatalysis. Anal. Chem..

[28] Kim J., Sim K., Cha S., Oh J.-W., Nam J.-M. (2021). Single-Particle Analysis on Plasmonic Nanogap Systems for Quantitative SERS. J. Raman Spectrosc..

[29] Khlebtsov N.G., Lin L., Khlebtsov B.N., Ye J. (2020). Gap-enhanced Raman tags: Fabrication, optical properties, and theranostic applications. Theranostics.

[30] Zhang Y., Ye J., Procházka M., Kneipp J., Zhao B., Ozaki Y. (2024). Gap-Enhanced Raman Tags (GERTs): Synthesis, Optical Properties, and Applications. Surface- and Tip-Enhanced Raman Scattering Spectroscopy: Bridging Theory and Applications.

[31] Li H., Tao K., Chen H., Zhang S., Sheng H., Fu L. (2025). Ultrasensitive and rapid screening of food allergy risk via aptamer-engineered AuMBA@AgNPs core-shell nanoparticle SERS tags-based lateral flow immunoassay. Sens. Actuators B Chem..

[32] Ferrari C.R., Moraes S.M., Buzalaf M.A.R. (2025). Saliva-based Hormone Diagnostics: Advances, applications, and future perspectives. Expert Rev. Mol. Diagn..

[33] Liu X., Yang X., Wang C., Liu Q., Ding Y., Xu S., Wang G., Xiao R. (2024). A nanogap-enhanced SERS nanotag–based lateral flow assay for ultrasensitive and simultaneous monitoring of SARS-CoV-2 S and NP antigens. Microchim. Acta.

[34] Wang W.-B., Li J.-J., Weng G.-J., Zhu J., Guo Y.-B., Zhao J.-W. (2023). An anisotropic nanobox based core-shell-satellite nanoassembly of multiple SERS enhancement with heterogeneous interface for stroke marker determination. J. Colloid Interface Sci..

[35] Kim J.-M., Lee C., Lee Y., Lee J., Park S.-J., Park S., Nam J.-M. (2021). Synthesis, Assembly, Optical Properties, and Sensing Applications of Plasmonic Gap Nanostructures. Adv. Mater..

[36] Eldridge B.K., Gomrok S., Barr J.W., Chaffin E.A., Fielding L., Sachs C., Stickels K., Williams P., Wang Y. (2023). An Investigation on the Use of Au@SiO_2_@Au Nanomatryoshkas as Gap-Enhanced Raman Tags. Nanomaterials.

[37] Sun P., Wang X., Song W., Chen L., Liu X., Yang L., Wang M., Liu R. (2025). Polydopamine engineered interfaces in metal-organic framework@plasmonic nanoparticles for improved SERS sensing. Sens. Actuators B Chem..

[38] Zhou J., Xiong Q., Ma J., Ren J., Messersmith P.B., Chen P., Duan H. (2016). Polydopamine-Enabled Approach toward Tailored Plasmonic Nanogapped Nanoparticles: From Nanogap Engineering to Multifunctionality. ACS Nano.

[39] Yilmaz M. (2019). Silver-Nanoparticle-Decorated Gold Nanorod Arrays via Bioinspired Polydopamine Coating as Surface-Enhanced Raman Spectroscopy (SERS) Platforms. Coatings.

[40] Wang D., Bao L., Li H., Guo X., Liu W., Wang X., Hou X., He B. (2022). Polydopamine stabilizes silver nanoparticles as a SERS substrate for efficient detection of myocardial infarction. Nanoscale.

[41] Paragliola R.M., Corsello A., Cera G., Locantore P., Piccirilli M., Salvatori R. (2026). Hypoprolactinemia: Biology, Clinical Relevance, and Diagnostic Challenges. Clin. Endocrinol..

[42] Khelifa L., Hu Y., Jiang N., Yetisen A.K. (2022). Lateral flow assays for hormone detection. Lab Chip.

[43] Shi L., Buhler E., Boué F., Carn F. (2017). How does the size of gold nanoparticles depend on citrate to gold ratio in Turkevich synthesis? Final answer to a debated question. J. Colloid Interface Sci..

[44] Yilmaz A., Yilmaz M. (2020). Bimetallic Core–Shell Nanoparticles of Gold and Silver via Bioinspired Polydopamine Layer as Surface-Enhanced Raman Spectroscopy (SERS) Platform. Nanomaterials.

[45] Bai T., Wang M., Cao M., Zhang J., Zhang K., Zhou P., Liu Z., Liu Y., Guo Z., Lu X. (2018). Functionalized Au@Ag-Au nanoparticles as an optical and SERS dual probe for lateral flow sensing. Anal. Bioanal. Chem..

[46] Qi X., Ye Y., Wang H., Zhao B., Xu L., Zhang Y., Wang X., Zhou N. (2022). An ultrasensitive and dual-recognition SERS biosensor based on Fe_3_O_4_@Au-Teicoplanin and aptamer functionalized Au@Ag nanoparticles for detection of Staphylococcus aureus. Talanta.

[47] Han C., Zhai W., Wang Y., Cao J., Wang M. (2022). A SERS aptasensor for rapid detection of aflatoxin B1 in coix seed using satellite structured Fe_3_O_4_@Au nanocomposites. Food Control.

[48] Rycenga M., Hou K.K., Cobley C.M., Schwartz A.G., Camargo P.H.C., Xia Y. (2009). Probing the surface-enhanced Raman scattering properties of Au–Ag nanocages at two different excitation wavelengths. Phys. Chem. Chem. Phys..

[49] Park S., Jung I., Lee S., Zhao Q., Lee S., Kim H., Park S. (2025). Au–Ag controllable composition nanoalloying of hexagonal nanoplates: Heterogeneous interfacial nanogaps enhance near-field focusing. Nanoscale.

[50] Hardy M., Chu H.O.M. (2025). Laser wavelength selection in Raman spectroscopy. Analyst.

[51] Lu L., Yu J., Liu X., Yang X., Zhou Z., Jin Q., Xiao R., Wang C. (2020). Rapid, quantitative and ultra-sensitive detection of cancer biomarker by a SERRS-based lateral flow immunoassay using bovine serum albumin coated Au nanorods. RSC Adv..

[52] Khlebtsov B., Pylaev T., Khanadeev V., Bratashov D., Khlebtsov N. (2017). Quantitative and multiplex dot-immunoassay using gap-enhanced Raman tags. RSC Adv..

[53] Cara E., Mandrile L., Sacco A., Giovannozzi A.M., Rossi A.M., Celegato F., De Leo N., Hönicke P., Kayser Y., Beckhoff B. (2020). Towards a traceable enhancement factor in surface-enhanced Raman spectroscopy. J. Mater. Chem. C.

[54] Pilot R., Bozio R. (2018). Validation of SERS enhancement factor measurements. J. Raman Spectrosc..

[55] Jin X., Zhang Y., Guo Q., Deng B., Tan Z., Liu F., Lin L., Ye J., Xu H. (2025). Multiplexed lateral flow immunoassays using high photostability gap-enhanced Raman nanotags: Highly sensitive, rapid, efficient and portable point-of-care tests. Biosens. Bioelectron..

[56] Serebrennikova K.V., Komova N.S., Barshevskaya L.V., Zherdev A.V., Dzantiev B.B. (2024). Highly sensitive SERS-based lateral flow immunoassay of fipronil using bimetallic Au@Ag@Ag nanorods. Microchim. Acta.

[57] Liebscher J. (2019). Chemistry of Polydopamine—Scope, Variation, and Limitation. Eur. J. Org. Chem..

[58] Whitehead S.J., Cornes M.P., Ford C., Gama R. (2015). Reference ranges for serum total and monomeric prolactin for the current generation Abbott Architect assay. Ann. Clin. Biochem..

[59] Zhong S., Liang H., Peng F., Lu Y., Liu T., Kulchytski U., Dong W. (2025). Simultaneous Detection of Prolactin and Growth Hormone Using a Dual-label Time-resolved Fluorescence Immunoassay. J. Fluoresc..

[60] Ortiz-Dosal A., Rodríguez-Aranda M.C., Ortiz-Dosal L.C., Núñez-Leyva J.M., Rivera-Pérez E., Cuellar Camacho J.L., Ávila-Delgadillo J.R., Kolosovas-Machuca E.S. (2024). Quasi-spherical silver nanoparticles for human prolactin detection by surface-enhanced Raman spectroscopy. RSC Adv..

[61] Sakthivel R., Lin L.-Y., Lee T.-H., Liu X., He J.-H., Chung R.-J. (2022). Disposable and cost-effective label-free electrochemical immunosensor for prolactin based on bismuth sulfide nanorods with polypyrrole. Bioelectrochemistry.

[62] Cai Q., Jin S., Zong H., Pei L., Cao K., Qu L., Li Z. (2023). A Quadruplex Ultrasensitive Immunoassay for Simultaneous Assessment of Human Reproductive Hormone Proteins in Multiple Biofluid Samples. Anal. Chem..

